# Supplementary material for: How do (perceptual) distracters distract?
Source: PLoS Comput Biol. 2022 Oct 13;18(10):e1010609. doi: 10.1371/journal.pcbi.1010609 (PMC9595561; doi:10.1371/journal.pcbi.1010609)
Supplement: S1 Appendix — (PDF) [file pcbi.1010609.s001.pdf]

S1 Appendix: Supplementary Analyses

I. Classic Signal Detection Theory parameters.

	**Exp.1**	**Exp.2**	**Exp.3**
Sensitivity, *d'*	1.59 (0.46)	1.41 (0.53)	1.7 (0.28)<br>*invalid*: 1.30(0.43)<br>*neutral:* 1.73 (0.38)<br>*invalid:* 1.90(0.30)
Bias, *c*	0.07 (0.15)	0.05 (0.15)	0.04 (0.09)<br>*invalid*: 0.03(0.12)<br>*neutral:* 0.06 (0.12)<br>*invalid:* 0.05(0.10)

We calculated sensitivity and bias following standard formulae (Green & Swets, 1966; Stanislaw & Todorov, 1999):

$$d' = -\varphi(H) - \varphi(FA)$$
$$c = -0.5\,(\varphi(H) + \varphi(FA))$$

where φ refers to the inverse cumulative function of the standard normal distribution (z-score), H refers to the number of hits (defined as the proportion of the time the participant responded clockwise when the target was tilted clockwise), FA refers to the number of false alarms (defined as the proportion of the time the participant responded clockwise when the target was tilted counterclockwise).

II. Psychometric functions.

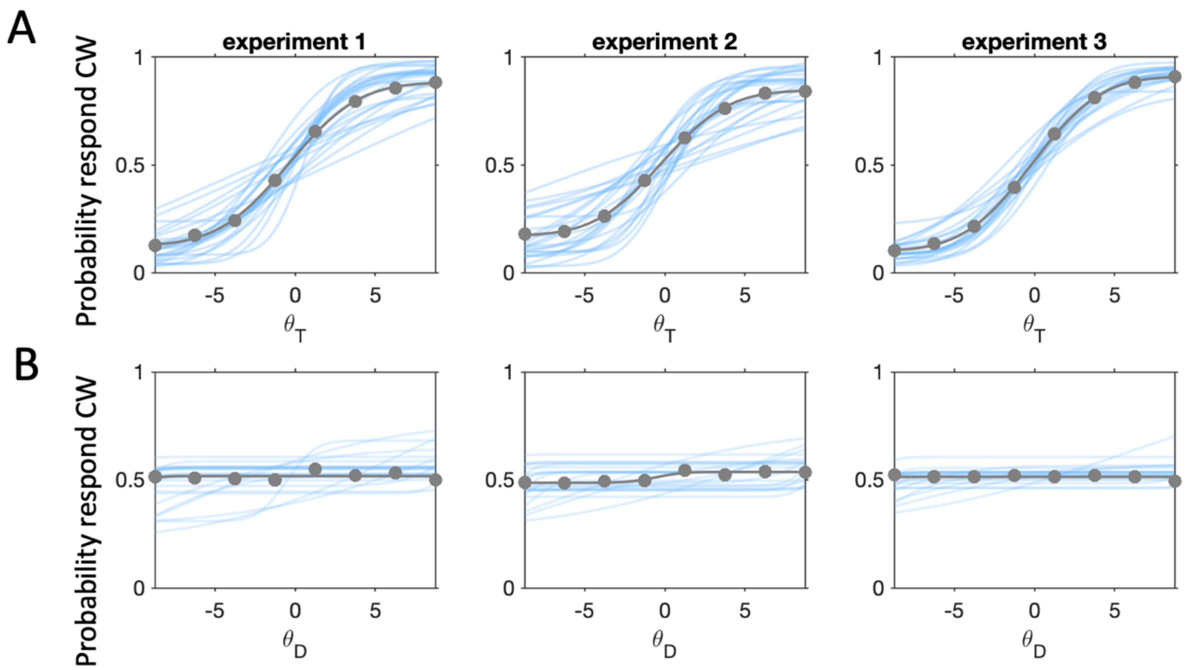

Panel A shows participant responses as a function of target tilt, θ_T. Panel B shows participant responses as a function of distracter tilt, θ_D. The three columns correspond to the three experiments. The light blue curves track psychometric functions for each individual participant. The grey curves correspond to a psychometric function fit to the aggregated data, collapsed across participants in each of the experiments; the grey dots correspond to data points.

All psychometric functions were estimated with the Psignifit toolbox for Matlab (Schütt et al., 2015). Stimulus orientation data was sorted into 8 equidistant bins. We fit cumulative Gaussian functions to categorization decisions for each participant individually (blue lines) and to aggregated data (grey lines).

III. Stepwise logistic regression.

We constructed a pool of putative predictor variables including the target and distracter features (independent effects) as well as interaction effects, informed by the literature reviewed in the introduction. The predictor variables we included correspond to:

- θ_T – the orientation of the target
- θ_D — the orientation of the distracter
- $|\theta_D|$ – the absolute value of the orientation of the distracter
- $congruency$ – a binary indicator whether the orientations of target and distracter fall on the same side of the category boundary or not
- $\theta_T \cdot \theta_D$ — the interaction between target and distracter orientations
- $\theta_T \cdot |\theta_D|$ — the interaction between the orientation of the target and the absolute value of the orientation of the distracter
- $\theta_T \cdot |\theta_T - \theta_D|$ — the interaction between target orientation and the consistency between the target and the distracter.

We used the stepwise generalized linear model fitting algorithm from the Statistics and Machine Learning Toolbox in Matlab, with a logistic link function and a model deviance-based cut-off criterion of 0.05 to add terms and 0.10 to remove terms. The plots below illustrate which terms were selected by the stepwise regression.

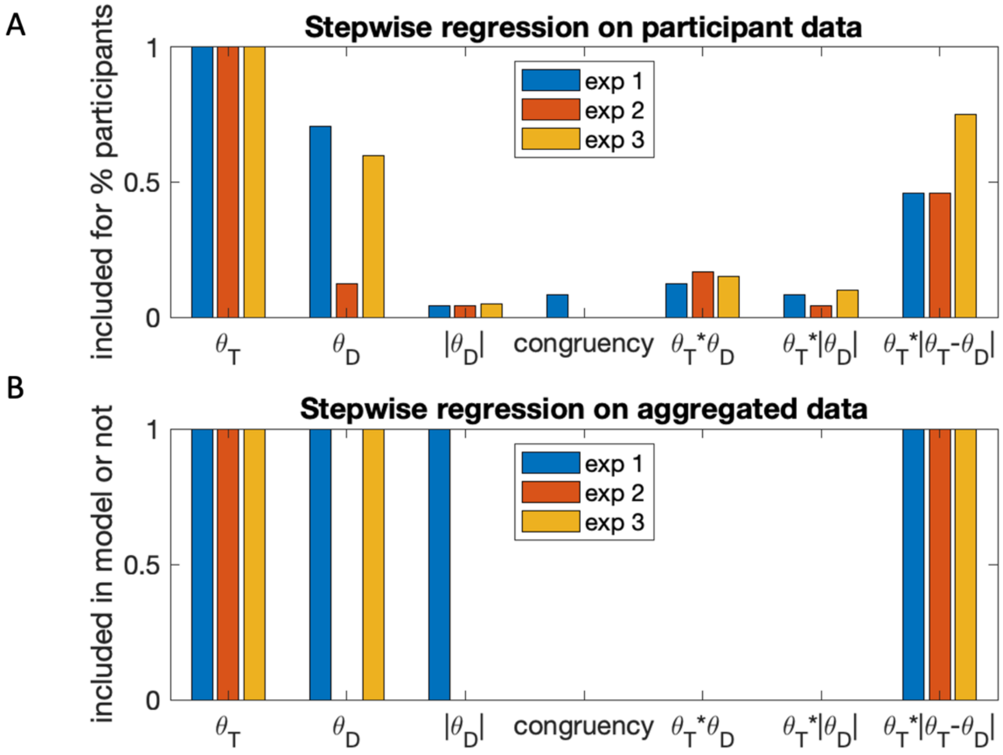

A. Results from stepwise regressions estimated individually for each participant in the three experiments. Bar height indicates the proportion of participants for whom parameter was included in final model. Bar colour indicates experiment number. **B.** Results from stepwise regressions estimated for aggregated data across all participants within an experiment. Bar heights indicate whether parameter included in final model or not. Bar colour indicates experiment number.

IV. Independent and interaction model fits.

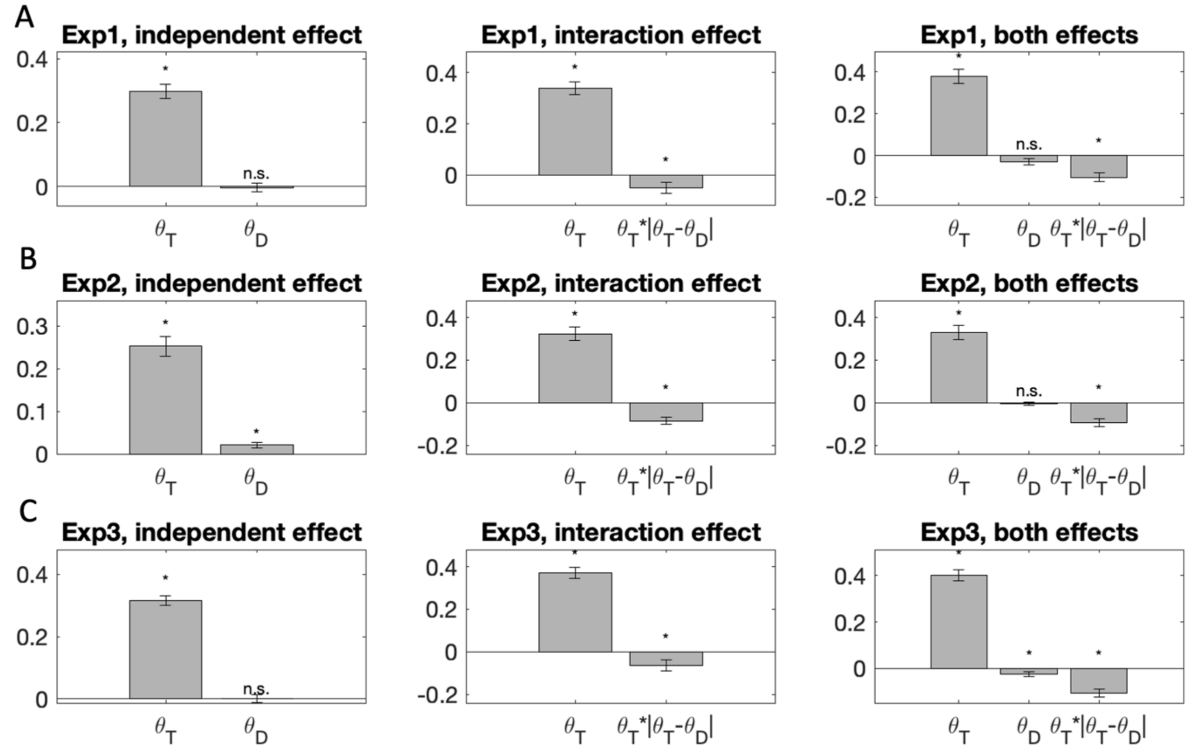

Independent and interaction models fit to human data, along with the combined model from the main text. Statistical significance (p<.05) of a t-test of each coefficient estimate across the participants is denoted by star (*). Across all experiments, the leftmost column corresponds to the independent effect model (θ_T and θ_D), the middle column corresponds to the interaction model (θ_T and $\theta_T \cdot |\theta_T - \theta_D|$), and the rightmost column corresponds to the combined model (θ_T, θ_D, and $\theta_T \cdot |\theta_T - \theta_D|$). **A.** Experiment 1. **B.** Experiment 2. **C.** Experiment 3.

V. Average stimulus energy profiles and ground truth kernels across the three experiments.

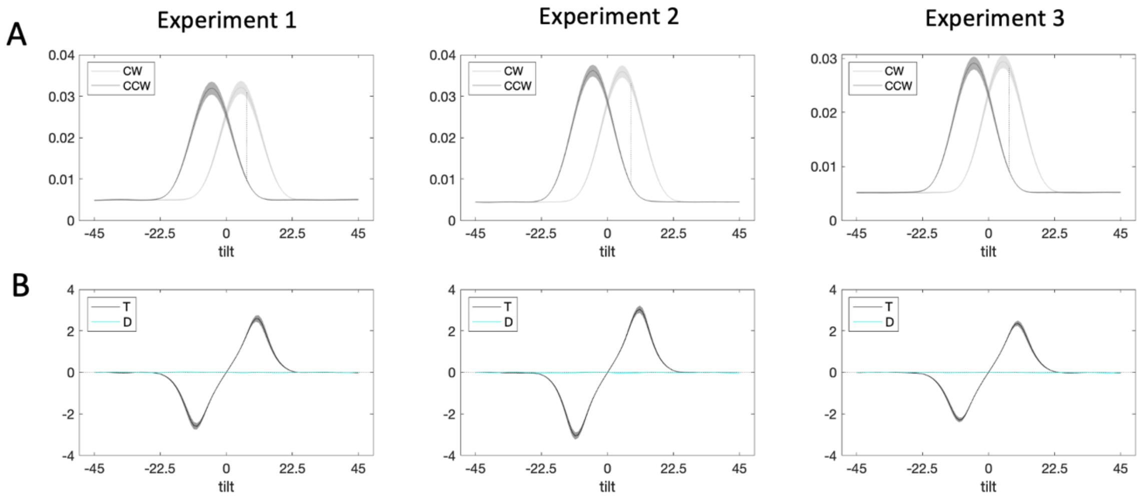

A. Average stimulus energy profiles (CCW=dark grey, CW=light grey; shaded regions correspond to M±SEM; the dashed line denotes maximum signal-to-noise ratio). **B.** Decision kernel based on ground truth stimulus tilts (target stimulus=black, distracter=cyan; shaded regions correspond to M±SEM).

VI. Statistical tests on decision kernels for the target and distracter stimuli.

	Near the boundary (< 22.5°)			Far from the boundary (> 22.5°)		
	t statistic	df	*p* value	*t* statistic	df	*p* value
**Target**						
Exp. 1	15.57	23	<.0001	1.78	23	.09
Exp. 2	13.06	23	<.0001	1.27	23	.22
Exp. 3	23.80	19	<.0001	-1.43	19	.17
**Distracter**						
Exp. 1	0.16	23	.87	-0.54	23	.59
Exp. 2	2.47	23	.02	0.17	23	.86
Exp. 3	-0.55	19	.59	0.09	19	.92

For statistical testing, we subtracted the counter-clockwise kernel estimates from the clockwise estimates (to place both decisions in a common frame of reference) and binned the data according to whether the orientation was near the boundary (< 22.5°) or far from the boundary (> 22.5°). We then tested each of these quantities against zero using t-tests.

Note that the distracter kernel in Exp.2 suggests a small direct attractive influence of the distracter. Perhaps counterintuitively, this pattern of results is actually in line with the group-level repulsive influences reported in Exp.1 and Exp.3. As analyses for Exp.2 are carried out on the tilt offset of the two stimuli relative to two orthogonal boundaries, inputs that are close in decision space (e.g. tilt offsets 10° and 9° = 1° distance in decision space) are actually further in sensory space (respectively 10° and 99° = 89° distance in orientation space) compared to inputs that are more distanced in decision space (e.g. tilt offsets 10° and -9° = 19° distance in decision space, respectively 10° and 81° = 79° distance in orientation space). Thus, to interpret these results in the same frame of reference as the findings from Exp.1 and Exp.3, we should swap the sign of the effect. The direct effect of the distracter does not emerge in any of the other analyses for Exp.2. This could perhaps be owing to the fact that the target-distracter tilt pairs used in Exp.2 were much more distant in orientation space compared to those in Exp.1 and Exp.3.

VII. Best fitting parameters for the normalization model.

	Exp.1	Exp.2	Exp.3
r	2.94 (1.88)	4.14 (3.02)	*invalid*: 4.1 (2.77)<br>*neutral*: 2.56 (1.86)<br>*valid*: 2.10 (0.92)
ρ	0.03 (0.51)	-0.05 (0.36)	0.12 (0.34)
τ	0.18 (0.19)	0.19 (0.17)	0.08 (0.05)

Values in table refer to mean and (standard deviation).

VIII. Feature swaps.

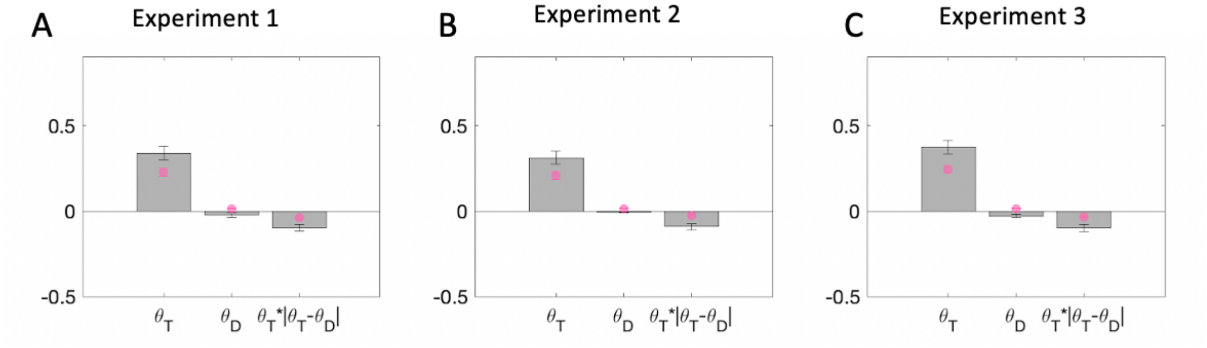

Here, we considered an alternative explanation for our data: that participants may confuse the two stimuli and swap the target and distracter features. To assess this account, we built a "noisy swaps" model controlled by two parameters: (i) the percentage of trials on which participant might swap the relevant stimulus and (ii) the slope of the choice function. We assessed model fit statistically using Bayesian model selection on cross-validated log likelihoods. We first fixed the number of swaps to 10%. This model is dispreferred relative to the normalization model (exceedance probabilities: Exp. 1, $p \sim 0$; Exp. 2, $p \sim 0$; Exp. 3, $p \sim 0$). Further, it fails to capture the qualitative signature of the data. In the figure above, the pink dots illustrate the regression estimates for data simulated with the swaps model. Swaps appear to produce a much weaker consistency bias than is observed in the human data (bars; A: Exp. 1, B: Exp. 2, C: Exp. 3 data collapsed across attention conditions).

Next, we relaxed the assumption that swaps happen 10% of the time, and instead estimated the proportion of time swaps occur as an additional free parameter λ. The average parameter value for λ we find is 0.003 ±0.01. That is, the model fitting exercise suggests that very few (if any) swaps occur in our data. Further, this model was again dispreferred relative to the normalization model (exceedance probabilities: Exp. 1, $p \sim 0$; Exp. 2, $p \sim 0$; Exp. 3, $p \sim 0$).

References:

Green, D. M., & Swets, J. A. (1966). *Signal detection theory and psychophysics* (Vol. 1). Wiley New York.

Schütt, H., Harmeling, S., Macke, J., & Wichmann, F. (2015). Psignifit 4: Pain-free Bayesian inference for psychometric functions. *Journal of Vision*, *15*(12), 474–474.

Stanislaw, H., & Todorov, N. (1999). Calculation of signal detection theory measures. *Behavior Research Methods, Instruments, & Computers*, *31*(1), 137–149. https://doi.org/10.3758/BF03207704
