# Supplementary material for: How do (perceptual) distracters distract?
Source: PLoS Comput Biol. 2022 Oct 13;18(10):e1010609. doi: 10.1371/journal.pcbi.1010609 (PMC9595561; doi:10.1371/journal.pcbi.1010609)
Supplement: Attachment — Submitted filename: Dumbalska et al 2022 review.docx [file pcbi.1010609.s002.docx]

The manuscript “How do (perceptual) distracters distract? “ of Dumbalska et al uses a set of 3 creative experiments the results of which are interpreted with regression models, psychophysical reverse correlation analyses and a divisive normalization computational model to better understand the question posed in the title. The authors center their work around 3 aspects of this question: 1) what is the nature of the influence (*independent vs interactive*) of the distractor on the perceptual decision about a target (binary categorization), 2) which information processing level does the distractor exert its influence (*perceptual level vs decision level*) and 3) how does *spatial attention* influence the answers to questions 1) and 2). Regression models are applied to address question 1, Experiments 1 and 2 together are able to address question 2 and experiment 3 uses spatial cues (neutral, invalid and valid 70% of the time) to address question 3. The reverse correlation analysis and divisive normalization model are used to confirm and further clarify these results: confirm the importance of *interactive* term between target value and the target to distractor difference to explain proportion correct in the binary categorization task, namely the *consistency bias* (in line with the author’s previous work Cheadle et al, 2014): the influence of the target on choices being higher when the target and distractor are consistent (close to each other in values, or otherwise having reduced *contextual variability*).

Overall, this paper represents a very nice formalization of how a distractor influences the categorization of a target, entails thorough analyses that build on each other and modeling. This paper is very well written, with intuitive explanations and nicely synthesized connections across literatures and approaches that are rarely all combined: *visual search, reverse correlation, divisive normalization modeling, spatial attention*. This work is important as it represents a novel and detailed application of the divisive normalization class of models (previously applied by the authors to other questions, Cheadle et al, 2014, Dumbalska et al, 2020) now to a task of visual search. I am very impressed and enthusiastic about this work and believe it will be a great paper and of high interest to the readers of PLOS Computational Biology. Before publication, I would like to ask the authors to provide some clarifications and additions. While the description of the disparate literatures covered is overall great and intuitive and very clear, at times more specific references, key terms and disambiguation are needed to help the readers. Several comments along this line are presented in the specific comments.

Specific comments.

1. Paragraph at lines 99-108. Consider including further citations: for instance, Palmer et al, 2000, Eckstein et al, 2011. I am not sure if it is entirely fair to say that the literature of visual search in general has been silent about how distractor information may influence or bias choices about the target itself – perhaps within the feature integration theory literature, yes. But is this not the case that in theory studies with identification tasks (i.e. Cameron et al, 2004) can address this question to some extent? One line of difference that I believe may help to be emphasized is the number of distractors (here 1, vs many of these studies, include much larger set sizes).
2. Paragraph at lines 124-139. Very nice connections to divisive normalization models. Please also include Navalpakkam and Itti, 2007 for a relevant approach.
3. Paragraph at lines 166-179. Could the authors explain a bit more the distinction between the distractor’s influence at the perceptual level vs decision level and the predictions of results of Exp. 1 and 2?
4. Lines 197-198 – Signal Detection theory analyses in Fig S1 and Table S1 –while they are standard, they could be elaborated more on in the methods – formula, etc/
5. Lines 200-201 – citations for the stepwise forward and backwards approach?
6. Line 219—the third term specifies the interaction in a form that we have previously called a “consistency bias”. Citation? I assume Cheadle et al, 2014 among others.
7. Line 414—computational modeling subtitle is perhaps not the most descriptive? Arguably the regression models before and psychophysical reverse correlation are also computational models, but not normative.
8. Paragraph at lines 483-49. Could the authors link this paragraph a bit better to the introduction? Not sure that it is fair to refer to the independent model by the standard model? Perhaps instead say independent model? Duncan and Humphreys’ 2009 and many other’s work on interactions between target and distractors in visual search may be related, though it is not immediately clear to me how.
9. Paragraph 495-501— different levels of distraction (meaning higher set sizes), vs in the author’s work. While Shen and Ma do not, Calder-Travis and Ma address the question of interactions between target and distractor. This is dissimilar from the interaction term in the regression models, but similar to what the tau term scales in the normalization model (what would be the min T-D difference in Calder-Travis and Ma, 2020). The search tasks are different (here binary categorization vs there detection (and localization)). I do not believe overlooking is thus quite fair to say; also do note task differences: a detection task of finding the target among distractors vs here binary categorization about the target. Also when writing tasks that broadly resemble our own - yes, they do, but somewhere may be useful to spell out binary categorization of target vs detection of target in a larger search array.
10. Lines 503-510….while this paragraph links this work (exp 3) to spatial cuing studies and Bayesian models with differential weighing, alludes to this through “variable decision-evidence”.
11. Lines 512-528—— line 513—citation — I assume it is Cheadle et al, 2014.
12. Figure 5. The connection with the consistency bias from the literature and the author’s previous work is great. Better word choice instead of static model (they already contrast in the text static models with diffusion models)? Perhaps just say parameters = 0. Why does figure 5 not show the parameters from the 3-parameter normalization model? May be helpful to also see them there.

References:

Palmer, J., Verghese, P., & Pavel, M. (2000). The psychophysics of visual search. Vision Research, 40(10- 12), 1227–1268. doi: 10.1016/s0042-6989(99)00244-8

Eckstein, M. P. (2011). Visual search: A retrospective. Journal of Vision, 11(5), 14–14. doi: 10.1167/ 11.5.14

Cameron, L. E., Tai, J., Eckstein, M., & Carrasco, M. (2004). Signal detection theory applied to three visual search tasks identification, yes/no detection and localization. Spatial Vision, 17(4), 295-325. doi: 10.1163/1568568041920212

Navalpakkam, V., & Itti, L. (2007, February). Search goal tunes visual features optimally. Neuron,

53 (4), 605–617. Retrieved from https://doi.org/10.1016/j.neuron.2007.01.018 doi: 10.1016/

j.neuron.2007.01.018
